# Downregulation of Barley *Regulator of Telomere Elongation Helicase 1* Alters the Distribution of Meiotic Crossovers

**DOI:** 10.3389/fpls.2021.745070

**Published:** 2021-09-30

**Authors:** Abdellah Barakate, Mikel Arrieta, Malcolm Macaulay, Sebastian Vivera, Diane Davidson, Jennifer Stephens, Jamie Orr, Miriam Schreiber, Luke Ramsay, Claire Halpin, Robbie Waugh

**Affiliations:** ^1^ Cell and Molecular Sciences, The James Hutton Institute, Dundee, United Kingdom; ^2^ School of Life Sciences, University of Dundee, Dundee, United Kingdom; ^3^ School of Agriculture and Wine, University of Adelaide, Waite Campus, Adelaide, SA, Australia

**Keywords:** barley, crossover, *RTEL1*, meiosis, recombination, RNAi

## Abstract

Programmed meiotic DNA double-strand breaks (DSBs), necessary for proper chromosomal segregation and viable gamete formation, are repaired by homologous recombination (HR) as crossovers (COs) or non-crossovers (NCOs). The mechanisms regulating the number and distribution of COs are still poorly understood. The regulator of telomere elongation helicase 1 (RTEL1) DNA helicase was previously shown to enforce the number of meiotic COs in *Caenorhabditis elegans* but its function in plants has been studied only in the vegetative phase. Here, we characterised barley *RTEL1* gene structure and expression using RNA-seq data previously obtained from vegetative and reproductive organs and tissues. Using RNAi, we downregulated *RTEL1* expression specifically in reproductive tissues and analysed its impact on recombination using a barley 50k iSelect SNP Array. Unlike in *C. elegans*, in a population segregating for *RTEL1* downregulated by RNAi, high resolution genome-wide genetic analysis revealed a significant increase of COs at distal chromosomal regions of barley without a change in their total number. Our data reveal the important role of RTEL1 helicase in plant meiosis and control of recombination.

## Introduction

Meiosis is a specialised cell division that generates haploid gametes through genome duplication followed by two rounds of chromosome segregation. At early prophase I, the homologs pair and synapse concomitantly with the introduction of a large number of DNA double-strand breaks (DSBs) by the topoisomerase type II-like protein Spo11 and its cofactors (Edlinger and Schlögelhofer, 2011; Panizza et al., 2011; Yadav and Bouuaert, 2021). The number of these programmed DSBs has been shown to be determined by the level of Spo11 activity (Xue et al., 2018) and restrained by the homolog engagement initiation during synaptonemal complex (SC) formation (Mu et al., 2020). Genome-wide mapping of meiotic DSB sites has been determined using chromatin immunoprecipitation followed by sequencing (ChIP-seq) and single-stranded DNA sequencing (SSDS; Khil et al., 2012) in several species including mouse (Lange et al., 2016), *Arabidopsis* (Choi et al., 2018), and maize (He et al., 2017). These studies revealed that the meiotic DSB landscape is tightly controlled by multi-factorial local DNA and chromatin epigenetic features.

The repair of meiotic DSBs by homologous recombination (HR) is initiated by exonucleolytic resection, a process recently shown to be controlled by ATM kinase in mouse (Yamada et al., 2020). Progressively, meiotic DSBs are repaired by a dynamic interplay of multiple recombinases resulting in different joint molecules (Wild et al., 2019). Resolvases act on these DNA structures to yield crossover (CO) and non-crossover (NCO) recombinants (San-Segundo and Clemente-Blanco, 2020). Only a small subset of the initial large number of meiotic DSBs are stepwise matured to form COs and their quasi-constant final number per meiocyte is species dependent and under tight homeostatic control (Cole et al., 2012). Although still poorly understood, the mechanisms of CO designation involve multiple factors ranging from the local genomic sequence to the chromatin structure (reviewed by Gray and Cohen, 2016; Dluzewska et al., 2018). Most eukaryotes including plants generate two types of COs: class I and class II that are sensitive or not to interference control, respectively (Wang et al., 2015). The majority of COs (class I) are produced through the so-called ZMM pathway and spaced out along the chromosomes, whilst non-interfering COs are on the Mus81-dependent pathway (Lynn et al., 2007; Shinohara et al., 2008; Osman et al., 2011; Zhang et al., 2019). The mechanism of interference is still unknown, although some advance has been made by the discovery that topoisomerase II (Top2) plays a key role in this CO distribution pathway in yeast (Zhang et al., 2014; Wang et al., 2015). However, in *Arabidopsis*, this topoisomerase was found to play a role in the resolution of meiotic chromosomal interlocks (Martinez-Garcia et al., 2018) whilst the CO interference is imposed by the SC (Capilla-Pérez et al., 2021; France et al., 2021).

Several CO antagonists have been identified in different forward genetic screens in *Arabidopsis*. Generally, genetic manipulation of these meiotic genes reduces fertility due to disturbance of the SC and/or meiotic recombination with changes in CO frequency and distribution. It has been widely proposed that fertile plants with increased recombination would be useful for crop improvement. In *Arabidopsis*, Crismani et al. (2012) identified suppressor mutations that restored fertility to a class of semi-sterile meiotic mutants in the ZMM pathway. They identified independent fertility restorers, including mutations in a conserved Fanconi anemia complementation group M (FANCM) helicase that had increased recombination (Crismani et al., 2012; Knoll et al., 2012) and FANCM-associated proteins MHF1 and MHF2 (Girard et al., 2014). The FANCM ortholog was found to direct NCO in competition with the pathway of class II COs (Lorenz et al., 2012). Several other fertility restorers in different meiotic genes and their interactors including RECQl4 (Séguéla-Arnaud et al., 2015), Top3 α (Séguéla-Arnaud et al., 2015, 2017), and FIGL1 (Girard et al., 2015; Fernandes et al., 2018a) have now been identified and shown to increase the overall recombination frequency in *Arabidopsis* (Séguéla-Arnaud et al., 2015; Fernandes et al., 2018a,b) and have been tested for their breeding utility in crops like Brassica (Blary et al., 2018), rice (Zhang et al., 2017; Fayos et al., 2019), and tomato (de Maagd et al., 2020).

In addition to FANCM and RecQl4, several other DNA helicases including the regulator of telomere elongation helicase 1 (RTEL1) are essential in ensuring genome stability during both vegetative and reproductive stages in plants (Dorn and Puchta, 2019). RTEL1 is a multifunctional DNA helicase essential for telomere maintenance, DNA replication, repair, and recombination; mutations in RTEL1 have been associated with several severe human diseases (Vannier et al., 2014). Its role as a suppressor of homologous recombination analogous to yeast SRS2 helicase was first reported in *C. elegans* and human cells (Barber et al., 2008; Villeneuve, 2008). The same group went on to demonstrate its function as an enforcer of meiotic CO interference and homeostasis in *C. elegans* (Youds et al., 2010). Genetic analysis of *C. elegans rtel1* mutants showed they lacked CO interference resulting in more COs in both chromosome centre and arm regions even though the number of DSBs was not affected. This work suggested that RTEL1 regulates meiotic recombination by dismantling transient strand invasions in D-loop joint intermediates and channelling DSBs repair by the synthesis-dependent strand annealing (SDSA) pathway promoting NCOs.

Based on the findings of Youds et al. (2010), *RTEL1* is therefore a potential target for improving crop breeding by altering patterns of recombination. However, its role in meiotic recombination in plants has not been reported. The two studies of *RTEL1* in plants were limited to the vegetative stage in *Arabidopsis* (Recker et al., 2014; Hu et al., 2015). These studies showed that RTEL1 helicase plays an essential role in safeguarding genome stability by repairing replication defects and telomere maintenance. Using a highly sensitive β-glucuronidase (*GUS*) based HR reporter system, it was demonstrated that RTEL1 helicase suppresses HR in somatic cells. Here, we studied the *RTEL1* gene structure and expression in barley (*Hordeum vulgare*) and downregulated its expression specifically in reproductive tissues of barley cultivar Golden Promise using an RNAi construct under the control of meiotic *DMC1* promoter. Selected RNAi lines were crossed to a different cultivar (cv. Bowman) and the derived F_2_ population analysed using a 50k iSelect SNP Array to determine at high resolution the impact of *RTEL1* downregulation on the landscape of meiotic recombination.

## Materials and Methods

### Plant Material

Barley (*Hordeum vulgare*) cultivars (cv.) Bowman (Bw) and Golden Promise (GP) and *RTEL1*^RNAi^ transgenics were grown in compost in a standard heated greenhouse as described by Barakate et al. (2014).

### Multiple Alignment and Phylogenetic Analysis

For RTEL1 phylogeny, orthologous sequences were identified from human (*Homo sapiens*), mouse (*Mus musculus*), African clawed frog (*Xenopus laevis*), Zebra fish (*Danio rerio*), *C. elegans*, fruit fly (*Drosophila melanogaster*), *Physcomitrella patens*, *Amborella trichopoda*, tobacco (*Nicotiana tabacum*), tomato (*Solanum lycopersicum*), soy bean (*Glycine max*), clementine (*Citrus clementina*), cassava (*Manihot esculenta*), *Arabidopsis thaliana*, *Brassica oleracea*, rice (*Oryza sativa*), maize (*Zea mays*), and pineapple (*Ananas comosus*) using OrthoFinder (v.2.3.3; Emms and Kelly, 2015). The longest orthologous sequences from each species were aligned using MAFFT (v7.266; Katoh and Standley, 2013). Alignments were refined using Gblocks (v0.91b; Castresana, 2000). Maximum likelihood phylogeny was computed using IQ-TREE (v1.6.9; Nguyen et al., 2015) using the LG+I+G4 substitution model and bootstrapping (*n*=100). The resultant phylogeny was plotted using FigTree (v1.4.3).

### RNAi Construct

A 750bp fragment corresponding to the 3′ end of *HvRTEL1* coding sequence was PCR-amplified using two *HvRTEL1* specific primers with Gateway sites (attB1-HvRTEL1 and attB2-HvRTEL1) and the full-length *HvRTEL1* cDNA plasmid as the DNA template. The obtained PCR fragment was cloned into the entry vector pDONR207 Gateway BP Clonase (Life Technologies). Using Gateway LR Clonase (Life Technologies), *HvRTEL1* fragment was then transferred into the destination vector pBRACT207m-HvDMC1p, a derivative of pBRACT207,^1^ where the maize ubiquitin 1 promoter was replaced by the barley DMC1 promoter. The final *HvRTEL1*^RNAi^ construct therefore has the RNAi cassette under the transcriptional control of the barley DMC1 promoter. As a control, a partial fragment of 715bp of β-glucuronidase (GUS) gene was cloned into the original pBRACT207 destination vector to make the construct GUS^RNAi^. A list of oligonucleotides used for plasmid construct preparation can be found in Supplementary Table 4.

### Preparation and Screening of Transgenic Plants

The strategy for regenerating and screening the transgenic plants is summarised in Supplementary Method 1. *HvRTEL1*^RNAi^ construct was transferred into *Agrobacterium tumefaciens* AGL1 strain containing the plasmid helper pSOUP. Barley cv. Golden Promise transformation was performed by the Functional Genomics Facility at The James Hutton Institute, Dundee, United Kingdom using immature embryos under hygromycin selection as described previously (Barakate et al., 2014; Colas et al., 2019). Hygromycin resistant T0 *HvRTEL1*^RNAi^ transgenic lines were grown in the glasshouse to maturity. Twenty T1 seeds per line were germinated on plates containing 0.5% phytagel in the presence of 100μg/ml of hygromycin (Jacobsen et al., 2006) to detect the presence of the transgene. Eight hygromycin resistant transgenic seedlings were transferred into 15cm pots of soil and grown in the glasshouse and their vegetative growth and fertility were monitored. In the T1 generation, fertility was scored for all transgenic lines and semi-sterile lines were selected for further analysis. Reduced fertility was used as a proxy for monitoring the impact of *RTEL1* down-regulation on meiosis as we demonstrated previously for several other meiotic genes in barley (Barakate et al., 2014; Colas et al., 2019).

### Crosses and Recombination Assays

T1 seeds of the selected lines were germinated in the presence of hygromycin and resistant seedlings with the *HvRTEL1*^RNAi^ transgene were grown in the glasshouse and crossed to Bowman cultivar. The obtained T_2_F_1_ seeds of the line *HvRTEL1*^RNAi^#11 were germinated in the presence of hygromycin, and resistant seedlings were grown in the glasshouse and the crosses were confirmed using Kompetitive Allele-Specific PCR (KASP™) assay (Rasheed et al., 2016) and F_1_ polymorphic markers (Supplementary Table 1). The resulting T_3_F_2_ seeds of Bw×*HvRTEL1*^RNAi^#11 were collected and used in KASP assay with F_2_ polymorphic markers (Supplementary Table 1) and 50K iSelect SNP Array (Bayer et al., 2017).

For KASP recombination assay, genomic DNA was extracted from glasshouse grown 2-week-old leaf tissue from 96 T_3_F_2_ individuals containing the *HvRTEL1*^RNAi^ transgene and 96 wild type (Bw×GP) F_2_ individuals using Qiagen QIAamp 96 DNA QIAcube HT kits on a QIAcube HT automated platform (Qiagen, Hilden, Germany). DNA quality was assessed using a Nanodrop 2000 (Thermo Scientific, Massachusetts, United States) with a requirement of >1.8 for 260/280 and 260/230 absorbance ratios. DNA was quantified using Picogreen (Thermo Scientific, Massachusetts, United States). Two independent *ca.* 10-cM intervals on 1H (centromeric) and 3H (distal) were studied using KASP assays (LGC, Biosearch, Hoddesdon, United Kingdom).

For 50K iSelect SNP Array based recombination assays, a total of 300ng of lyophilised DNA was sent to Geneseek (Neogen Corporation, Auchincruive, United Kingdom) for Illumina HTS processing and HiScan chip imaging (Illumina, San Diego, United States). SNP R and Theta values were extracted using GenomeStudio Genotyping Module v2.0.2 (Illumina, San Diego, California) and allele scores were created using paRsnps (an in-house software package for clustering, visualising and comparing Illumina SNP genotyping data).

### Recombination Data Analysis

#### Data cleaning

Genotypic data were cleaned with a custom R script which filtered out monomorphic and low-quality markers. Similarly, individuals with excessive missing or low-quality data were also removed from the analysis.

### 50K Recombination Analysis

The physical order of the markers and their respective physical positions on the barley genome were obtained from the current physical assembly (MorexV2, Monat et al., 2019). Crossovers were counted as the change of allele between two consecutive markers, from either a parental allele to heterozygous or the other way around. Double recombination events were counted with a score of 2 when the allele call was switching from one parental homozygous genotype to the other. To ensure the number of crossovers was not inflated by the isolated allele switches produced by genotyping errors, the changes of alleles were only counted as valid if they were maintained in the following and previous three markers. The effect of missing data in the three-marker window on CO calling was corrected manually.

The recombination fraction (r) was calculated by dividing the number of crossovers between consecutive markers by the number of individuals that had data for those markers. Finally, the genetic distance between two markers was calculated by using the Kosambi formula as follows:

$$\begin{array}{l} Kosambi\;genetic\;distance\;\left(cM\right)=\frac{\ln\left[\left(1+2*\left(\frac{r}{2}\right)\right)/\left(1-2*\left(\frac{r}{2}\right)\right)\right]}{4}*100 \end{array}$$

Recombination differences were compared using the Wilcoxon’s signed rank test (as published in Devaux et al., 1995) using the R ggpubr package.

## Results

### Barley *RTEL1* (*HvRTEL1*) Gene Structure and Expression

A BLAST search was performed with the *Arabidopsis RTEL1* amino acid sequence (AT1G79950.1) to find the rice *RTEL1* mRNA sequence (Os01g0592900). This monocot mRNA sequence was then used in a BLAST search of our inhouse transcriptome database of meiotic anthers of barley cv. Optic and one partial cDNA sequence (TrinityTranscript16036) of 2,393bp was found. Barley *RTEL1*-specific primers were used in rapid amplification of cDNA ends (RACE) and reverse transcription PCR (RT-PCR) to clone the full-length cDNA of 3,370bp (NCBI accession number: MW689197). This cDNA is comprised of 2,964bp of open reading frame (ORF) flanked by 88bp and 318bp corresponding to the 5′-UTR and the 3′-UTR, respectively. Like other barley meiotic genes that we have characterised (Higgins et al., 2012; Colas et al., 2016, 2019; Barakate et al., 2021), a high GC content of up to 80% was detected at the 5′ region in a GC scan of the transcript BAnTr.GP.3HG008068.1 using a 50bp window (data not shown).

**Figure 1:**
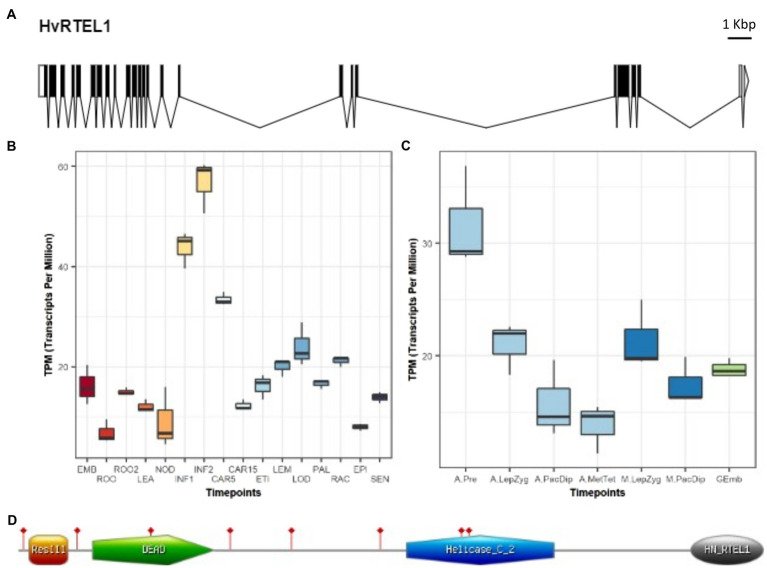
Barley regulator of telomere elongation helicase 1 (*RTEL1*) gene structure and expression. **(A)** Gene structure showing exons (black boxes), introns (black lines), and 5′ (white box) and 3′ (clear box and arrowhead) UTRs. **(B)** Expression levels in different parts and developmental stages of barley. The samples (three replicates each) are: EMB, 4-day embryos dissected from germinating grains; ROO, Roots from the seedlings (10cm shoot stage); ROO2, Root (4weeks); LEA, Shoots from the seedlings (10cm shoot stage); NOD, Developing tillers at six leaf stage, 3rd internode; INF1, Young developing inflorescences (5mm); INF2, Developing inflorescences (1–1.5cm); CAR5, Developing grain, bracts removed (5 DPA); CAR15, Developing grain, bracts removed (15 DPA); ETI, Etiolated (10day old seedling); LEM, Lemma (6weeks PA); LOD, Lodicule (6weeks PA); PAL, Palea (6weeks PA); RAC, Rachis (5weeks PA); EPI, Epidermis (4weeks PA); and SEN, Senescing leaf (2months). **(C)** Expression levels in barley anthers and meiocytes at different developmental stages. The samples (three replicates each) are: A.Pre, anther pre-meiosis; A.LepZyg, anther leptotene–zygotene; A.PacDip, anther pachytene–diplotene; A.MetTet, anther metaphase I–tetrad; M.LepZyg, meiocyte leptotene–zygotene; M.PacDip, meiocyte pachytene–diplotene, and Gemb, germinating embryos. The prefixes A., and M. in the sample names depict anther and meiocyte samples, respectively. **(D)** RTEL1 protein sequence (grey line) contains four conserved pfam domains (coloured shapes). The sites of eight conserved MEME motifs are flagged in red.

A barley genomic contig of 34.464 Kbp that contained the entire *RTEL1* gene was found (RTEL1.mrna1_gpv1_chr3H_317419392_317444304_1000) in the barley Golden Promise genomic database (Schreiber et al., 2020). The structure of barley *RTEL1* (*HvRTEL1*) was determined by alignment of the full-length cDNA and gene sequences (Figure 1A). *HvRTEL1* contains 23 exons and 24 introns including two in the 3′-UTR. Interestingly, the *HvRTEL1* gene contains two large introns in the coding sequence (Intron 16: 6.690Kbp and Intron 19: 10.744Kbp) and one at the 3’-UTR (Intron 23: 4.140Kbp).

Based on transcriptomic data from 16 different tissues and developmental stages of barley (International Barley Genome Sequencing Consortium, 2012), *RTEL1* is most highly expressed in young inflorescences compared to other vegetative tissues (Figure 1B). Expression was also detected in the transcriptomic data of isolated barley anthers and meiocytes (Barakate et al., 2021). Although *RTEL1* is expressed during all meiotic stages (and in germinating embryos), the highest transcript level was detected in anthers at the pre-meiosis stage (Figure 1C). In addition, a total of eight predicted *RTEL1* transcript isoforms were found in this Golden Promise barley anther and meiocyte transcriptomic data. Except for the isoforms 1 and 4 that are full-length (2,964nt coding sequence), all six transcripts were alternatively spliced. Isoform 3 resulted from a partial exclusion of exon 10, an alternative splicing (AS) event that was detected in all analysed samples of anther and meiocyte developmental stages and germinating embryos. A similar partial exclusion of exon 12 was found in the transcript 6. In isoform 2, only the first 14 introns were spliced out whilst isoform 8 retained the first 5 introns. Isoform 5 retained 111nt of intron 17 and isoform 7 was missing 10nt of exon 21. Consequently, an ORF was maintained of only four transcripts (1, 3, 4, and 6). With the exception of transcript 4 (BAnTr.GP.3HG008068.4), the remaining seven isoforms showed negligible expression in all samples (Supplementary Figure 1). *HvRTEL1* encodes a protein of 987 amino acid residues with a predicted MW of 110.5kDa and an overall pI of 8.78. HvRTEL1 contains ResIII (15–67 aa), DEAD_2 (99–261 aa), Helicase_C_2 (515–710 aa), and Harmonin_N_Like (891–987 aa) pfam domains as previously reported for its *Arabidopsis* and mammalian orthologues (Recker et al., 2014; Figure 1D). A phylogenetic tree shows clear separation of the monocotyledon and dicotyledon RTEL1 sequences with the barley protein closest to those of wheat and goat grass (Supplementary Figure 2). The Plant RTEL1 protein sequences used to build the phylogenetic tree are relatively well conserved. Their analysis with GLAM2 online software (Frith et al., 2008) revealed eight highly conserved motifs with the best at 480–534 aa of the protein sequence (Figure 1D). None of them overlaps with the AS event of exon 10. Analysis of natural variation in barley (Russell et al., 2016) detected three synonymous and one nonsynonymous (D750E) variant, a conserved amino acid in monocotyledons. According to cultivar Morex genome assembly version 2, barley chromosomes are divided into three zones: zone 1 (distal) enriched in rapidly evolving genes, zone 2 (interstitial) and zone 3 (proximal) enriched in housekeeping genes (Mascher et al., 2017). The *RTEL1* gene is on chromosome 3 (343,594,707–343,619,631bp) close to the centromeric end of interstitial Zone 2 where recombination is low.

### Suppression of Barley RTEL1 During Meiosis Affects Plant Fertility

To avoid potential negative impact of constitutive downregulation of *RTEL1* on vegetative growth of barley transgenics, such as the stunting phenotype previously reported for barley *ZYP1* (Barakate et al., 2014) and *MSH7* (Lloyd et al., 2007) genes, the maize ubiquitin 1 promoter in the transformation vector pBRACT207 was replaced with the barley DMC1 promoter to drive the expression of *RTEL1* RNAi specifically in spikes and meiocytes (Colas et al., 2019). Stunted vegetative growth phenotypes were absent for transgenics containing a *GUS*^RNAi^ construct, where a fragment of *E. coli* β-glucuronidase (*GUS*) was used as a control. A total of 23 T0 independent *HvRTEL1*^RNAi^ lines were recovered in barley cv. Golden Promise and transgene integration in T0 plants was confirmed by PCR using their genomic DNA and a pair of oligonucleotides specific to the selectable hygromycin marker gene. Six T0 lines were completely sterile and the number of seeds of the remaining 17 T0 lines were scored and compared to the control *GUS*^RNAi^. Whilst *HvRTEL1*^RNAi^ lines showed different degrees of semi-sterility, 23 regenerated *GUS*^RNAi^ lines were fully fertile producing an average of 22.4±5.5 seeds per tiller (Supplementary Figure 3A). This result is in agreement with the previous study showing semi-sterility of *RTEL1* T-DNA knockout in *Arabidopsis* (Röhrig et al., 2016). Twenty T1 seeds from each of *HvRTEL1*^RNAi^ and *GUS*^RNAi^ lines were germinated in the presence of hygromycin and eight resistant T1 seedlings per line were grown to maturity and screened for reduced fertility as an indicator of a possible meiotic defect. In total, seven *HvRTEL1*^RNAi^ lines that consistently showed reduced fertility in all of their corresponding T1 plants compared to the control were selected for further analysis (Supplementary Figure 3B). The seeds of the *HvRTEL1*^RNAi^#8 line were very thin and did not germinate. The *HvRTEL1*^RNAi^#9 line was also excluded since 50% of its plants produced very few seeds (0–28). Four lines (*HvRTEL1*^RNAi^#1, *HvRTEL1*^RNAi^#2, *HvRTEL1*^RNAi^#11, and *HvRTEL1*^RNAi^#15) were selected and fertilised using pollen from cultivar Bowman and their F_1_ seeds collected. The reciprocal cross was not performed as it was shown to be less successful in *Arabidopsis rtel1* mutant (Röhrig et al., 2016). Untransformed barley Golden Promise was also crossed to Bowman to provide a (GP×Bw) control. True F_1_ progenies were confirmed using Kompetitive Allele-Specific PCR (KASP™) assays (Rasheed et al., 2016) for specific SNPs on barley chromosomes 2H and 7H (Supplementary Table 1). Since *HvRTEL1*^RNAi^#11 was less fertile than the other three lines, its corresponding F_1_ seeds were selected in the presence of hygromycin to maintain the transgene and their F_2_ seeds collected. A total of 18 F1 lines were generated and F_2_ seeds of a single F_1_ individual *HvRTEL1*^RNAi^#11×Bw.1 (*RTEL1*^RNAi^×Bw.1) were chosen to assess the effect of *RTEL1* down-regulation on meiotic recombination.

### Downregulation of RTEL1 Increases Recombination at Sub-Telomeric Regions

A total of 96 F_2_ seeds were germinated for both *RTEL1*^RNAi^×Bw.1 and GP×Bw.1 and their genomic DNA extracted. A preliminary assessment of recombination rate was performed using KASP assays for SNP markers that bounded the 1H pericentromeric and 3H distal chromosomal regions (Supplementary Table 1). Compared to GP×Bw.1 control, *RTEL1*^RNAi^×Bw.1 line showed a reduction (from 6.90 to 3.30%) and increase (from 7.70 to 14.50%) of recombination in 1H pericentromeric and 3H distal regions, respectively (Supplementary Table 2). This pattern of recombination was probed further using the barley 50k iSelect SNP Array (Bayer et al., 2017). From the initial 43,799 markers, 11,125 were used for analysis after filtering out all monomorphic and low-quality markers. Twelve individuals out of 192 samples were removed from the analysis for reasons of quality control as their data revealed a high number of missing data and heterozygous calls. This left 90 individuals per population for the genetic analysis.

### 50K Polymorphism Coverage

There was an average of 1,589 SNP loci per chromosome (Supplementary Table 3) with chromosome 1H having considerably fewer (772) compared to the rest. Following the genomic zone distribution described by Mascher et al. (2017), most loci were in the interstitial Zone 2 (61.2%), which is the largest physical zone, with the distal Zone 1 containing 29.1% of the SNP loci, whilst very few were found in the centromeric Zone 3 (3.5%). The overall density of markers per chromosome showed that the distal areas (Zone 1), despite having a smaller number of markers, had the densest coverage (9.1 SNP/Mbp) compared to the interstitial areas (Zone 2) with 2.5 SNP/Mbp, whilst zone 3 had in total 0.4 SNP/Mbp.

The positions of the first and last loci on each chromosome were verified to determine the potential missing coverage of the genetic map and very distal CO (Supplementary Table 3). The largest uncovered regions were the short arms of chromosomes 5H, 6H, and 7H with 0.33, 0.26, and 0.24% of the physical chromosome not covered, respectively, and the long arms of chromosomes 3H and 4H with the terminal 1.1 and 0.35% missing, respectively.

### Recombination Frequency

The overall correlation of genetic and physical maps is similar in both GP×Bw.1 and *RTEL1*^RNAi^×Bw.1 populations. However, the control F_1_ cross showed higher total recombination frequency compared to the *RTEL1*^RNAi^×Bw.1 line with a total genetic map of 1034.3cM compared to 987.6cM. This would correspond to 20.6 and 19.7 COs in the control and *RTEL1*^RNAi^×Bw.1 populations, respectively (assuming 50cM/CO). However, this difference of 46.7cM is not significant according to Wilcoxon’s signed rank test. At the chromosome level, the control population showed higher recombination in all chromosomes except for the chromosomes 2H and 5H where recombination was higher in *RTEL1*^RNAi^×Bw.1 line.

### Recombination Distribution

**Figure 2:**
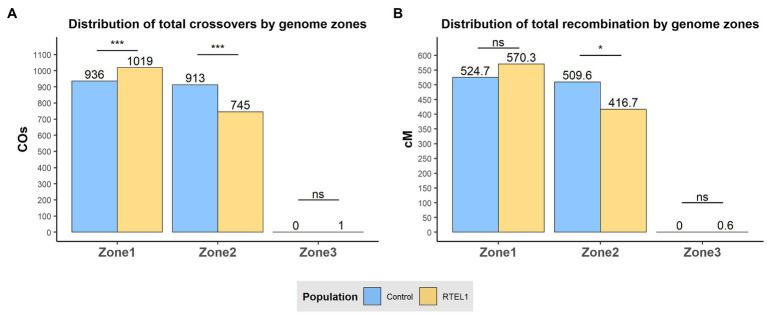
Effect of *RTEL1* downregulation on meiotic recombination and crossover events at sub-chromosomal level. Genome-wide distribution of crossover events **(A)** and recombination **(B)** by genomic zone for the control GP×BW (blue) and RTEL1^RNAi^×BW (yellow) populations. The genomic physical limits between the genomic zones are described in Mascher et al. (2017). Significance in **(A)** refers to the comparison with the χ-squared test of the proportion of crossovers and in **(B)** to the Wilcoxon’s signed rank in each zone between population tests. ns=not significant; ^*^ *p*<0.05; and ^***^ *p*<0.001.

Analysis of the recombination distribution was initially carried out by utilising the divisions of the chromosomes into the three genomic zones described by Mascher et al. (2017). The three genomic zones were defined based on the median of 20-mer frequencies, Zone 1 being characterised by distal regions high in gene content and low-copy elements; Zone 2 by large interstitial regions with intermediate gene-density and high 20-mer elements; and Zone 3 by proximal regions, with unique conserved sequences and low recombination (Mascher et al., 2017). At a genome-wide level, across all chromosomes, *RTEL1*^RNAi^×Bw.1 population showed higher recombination (570.3cM) than the control (524.7cM) in the distal Zone 1 although this was not significantly different. However, for Zone 2 the *RTEL1*^RNAi^×Bw.1 population exhibited significantly lower (*p*<0.05) recombination than the control with totals of 416.7 and 509.6cM, respectively. Zone 3 exhibited practically no recombination in either population, with the *RTEL1*^RNAi^×Bw.1 only exhibiting 0.6cM for this region. The relative change of crossover distribution of the *RTEL1*^RNAi^×Bw.1 population from Zone 2 to Zone 1 was tested with a χ-squared test by comparing the proportions of crossovers between the two populations for every zone with a contingency table (Figure 2A). Zones 1 and 2 showed significant differences (*p*<0.001) in the relative change of distribution of crossovers between populations, as *RTEL1*^RNAi^×Bw.1 population showed a relatively higher proportion of crossovers in Zone 1 compared to Zone 2 (57.7/42.2%), whilst the control had a similar proportion of crossovers in both zones (50.6/49.4% respectively). At the chromosomal scale, this pattern in Zone 2 (less recombination in the *RTEL1*^RNAi^×Bw.1 population) was maintained for all chromosomes, whilst the higher recombination in Zone 1 of *RTEL1*^RNAi^×Bw.1 was detected in all chromosomes except 4H and 6H (Figure 2B; Supplementary Figure 4).

**Figure 3:**
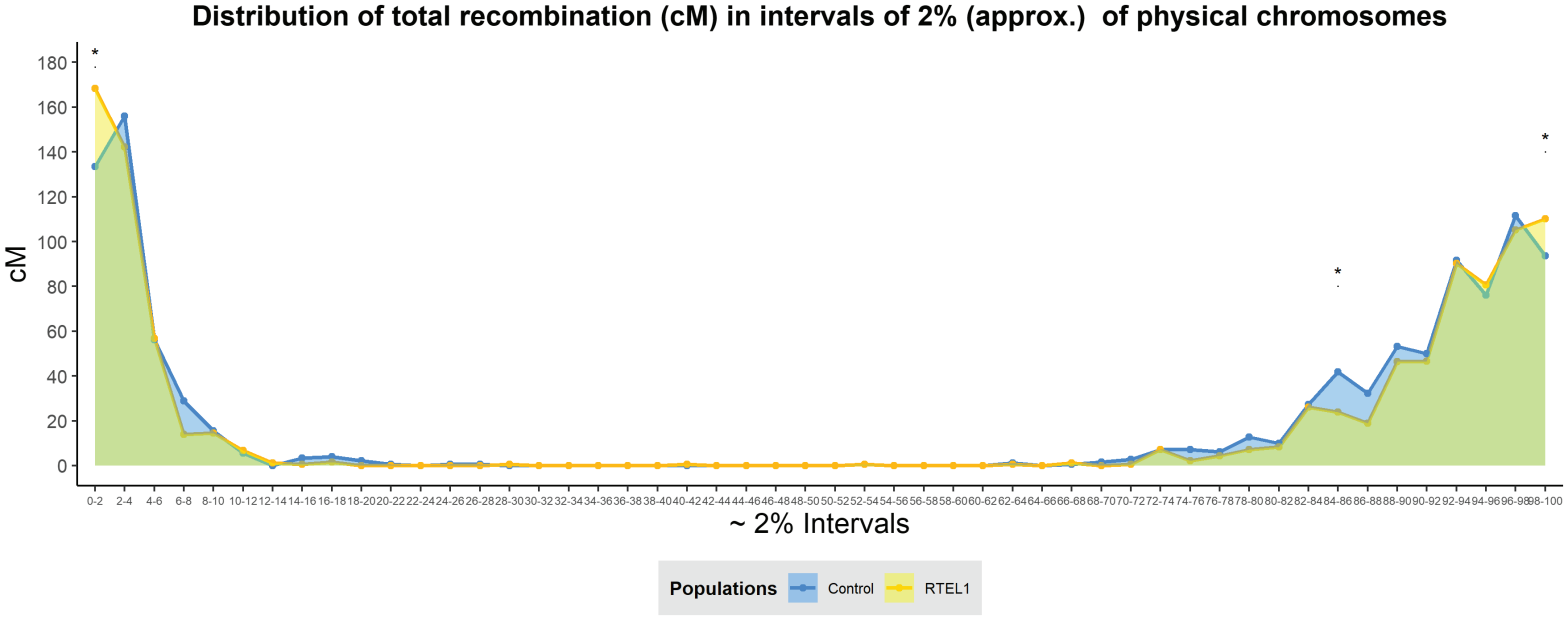
Effect of *RTEL1* downregulation on total meiotic recombination distribution at 2% chromosomal windows. Genome-wide recombination distribution by intervals of physical 2% for the GP×BW (blue) and *RTEL1*^RNAi^×BW (yellow) populations. The differences between populations were tested for every interval with the Wilcoxon’s signed rank test being ^*^ *p*<0.05. The green area under the lines shows the shared recombination regions by both populations, whilst blue or yellow show regions where only the control or *RTEL1*^RNAi^ populations recombine, respectively.

The differences in recombination distribution were also analysed by dissecting the genome into physical intervals of 2% of each chromosome, allowing a Wilcoxon’s signed rank test to be carried out. The distribution changes were visualised by merging all the chromosomes together dividing the genome in 50 bins (Figure 3). This analysis allowed a higher resolution of recombination distribution, showing the increase of the distal recombination in *RTEL1*^RNAi^×Bw.1 compared to the control was located in the most distal 2% in both the short (increase of 34.8cM, *p*<0.05) and the long arms (16.6cM, *p*<0.05) of the chromosomes. Similarly, the recombination levels in the interstitial regions were significantly lower (17.8cM, *p*<0.05) in the 84–86% interval in the long arms. This increase in recombination in the distal regions is not evenly shared between chromosomes (Figure 2A), with the short arms of chromosomes 2H and 6H and the long of 5H contributing the most, with increases of 10, 7.9, and 10.6cM, respectively.

## Discussion

Meiotic recombination, which is necessary for proper chromosomal segregation, is tightly regulated by the intricate integration of programmed DSB induction, HR repair machinery and chromosomal remodelling resulting in the right proportions of CO and NCO recombinants (Altendorfer et al., 2020). The frequency and distribution of COs are regulated by the still poorly understood phenomena of CO homeostasis and interference, respectively (Wang et al., 2015). Understanding the molecular mechanisms that limit the number and close spacing of COs is of interest not least for the potential applications in crop breeding (Taagen et al., 2020). Several DNA helicases including RTEL1 have been found to dismantle most of the D-loop joint molecules of recombination channeling their repair through the NCO pathway (Dorn and Puchta, 2019). Given its importance in genome stability and its relevance to human health (Vannier et al., 2014), RTEL1 helicase has been well studied in animals including its role in meiotic CO homeostasis and interference imposition in *C. elegans* (Youds et al., 2010). Surprisingly, studies of this important helicase in plants have been limited to the vegetative stage in *Arabidopsis* (Recker et al., 2014; Hu et al., 2015; Röhrig et al., 2016; Olivier et al., 2018; Dorn et al., 2019; Aklilu et al., 2020). In this study, we characterised the barley *RTEL1* gene structure and expression and downregulated its expression in reproductive tissues using RNAi to determine its function in meiotic CO frequency and distribution.

The barley RTEL1 helicase is encoded by a single gene that contains 24 introns including two large ones in the coding sequence and one in the 3′-UTR. We have found such large introns in other genes involved in meiosis such as *FIGL1*, *HEI10*, *KU70*, *Met1A*, and *MSH2* but their significance remains to be determined. It is possible that they could relate to gene expression regulation as reported in Drosophila where large introns can promote alternative splicing (Kandul and Noor, 2009) although only 16 such large introns (>5kbp) were found in *Arabidopsis* (Chang et al., 2017). Although we detected eight *RTEL1* transcripts in barley anther and meiocyte transcriptome (Barakate et al., 2021), only one isoform was expressed. Interestingly, we detected several AS events of partial exon skipping (exon 10, exon 12, and exon 21) and intron retention in all samples. Such AS events were also detected for *Arabidopsis RTEL1* gene (Martín et al., 2021) but remain to be confirmed by isoform specific PCR amplification and sequencing. These AS events would be of potential interest to detect and quantify at tissue level resolution to determine whether they are involved in the regulation of *RTEL1* expression in plants. Indeed, AS and in particular intron retention was shown to control the timely usage of transcripts during meiosis in mice (Naro et al., 2017) and Brassica (Golicz et al., 2021). RTEL1 expression was found to be highest in spikes compared to other barley tissues and developmental stages and highest in isolated anthers at the pre-meiosis stage, concurrent with its known function in DNA replication. Its expression in isolated meiocytes indicates a potential function in meiotic recombination as previously shown in *C. elegans* (Youds et al., 2010).

We generated barley transgenic plants, where *RTEL1* was downregulated specifically in reproductive tissues using a previously described barley DMC1 promoter thus avoiding potential deleterious effects on vegetative growth (Colas et al., 2019). Although half of the T0 lines were completely sterile, other lines maintained semi-sterility in the T1 generation and enabled the development of a mapping population for recombination analysis. The genetic map generated for the control population GP×Bw (1034.3cM) approximates other barley genetic maps calculated in previous studies including for GP×Morex population (Bayer et al., 2017). Although the overall number of COs and total genetic map was not significantly altered by *RTEL1* downregulation, the density of marker coverage used allowed the detection of subtler changes of recombination distribution. At a genomic zone delimitation-scale (Mascher et al., 2017), *RTEL1* downregulation showed significantly less recombination in the interstitial Zone 2 and non-significant but higher recombination in the distal Zone 1 compared to the control. Interestingly, when dissecting the genome into physical intervals of 2%, the increase of distal recombination under the *RTEL1* downregulation was significant and concentrated mainly in the first and last 2% physical intervals of the genome. This increase of recombination was attributed mainly to chromosome 2H, 6H short arm, and 5H long arm, but given the extremely distal nature of it, it would be assumable that recombination in these regions is likely to be underestimated and highly affected by the lack of polymorphism in the very distal ends of chromosome 3H, 4H, 5H, and 6H (Supplementary Table 3). It is potentially of relevance that RTEL1 inhibits HR during replication and at telomere ends (Dorn and Puchta, 2019), which could align with this very distal increase in recombination detected when downregulating the gene.

The results suggest that the change of distribution is more likely to be driven predominantly by a shift of recombination, skewing the distribution from Zone 2 towards Zone 1 regions under the *RTEL1* downregulation, rather than an absolute increase and reduction of recombination. Alterations in the timing of synapsis could play a role in this shift as this has been previously related to crossover distribution changes in barley (Higgins et al., 2012), though further studies using immunocytology would be necessary to correlate the spatiotemporal binding of RTEL1 with DSB repair and CO distribution and chromosome pairing. It will be interesting to confirm whether the newly discovered end-adjacent regions (EARs) in yeast (Subramanian et al., 2019) are also present in plants and their potential contribution to CO increase in the *RTEL1*^RNAi^ population.

The overall pattern of recombination in the barley *RTEL1*^RNAi^ line was different from that in a *C. elegans* knockout mutant where the CO number and distribution were strongly altered (Youds et al., 2010). This potentially is due to differences in the mutant as well as the recombination landscape and control in the two species.

Earlier studies of RTEL1 in plants demonstrated its important role in genome stability during vegetative growth of *Arabidopsis* (Recker et al., 2014; Hu et al., 2015). RTEL1 was found to play essential roles during replication-associated DNA damage, stabilising repetitive elements and rDNA repeats and antagonising homologous recombination (Röhrig et al., 2016; Dorn et al., 2019; Dorn and Puchta, 2019). This function seems to be conserved in plants as shown in *Physcomitrella patens* (Kamisugi et al., 2016; Goffová et al., 2019). Using a terminal restriction fragment assay (TRF) in *Arabidopsis*, Hu et al. (2015) concluded that RTEL1 loss does not result in telomere shortening whilst Recker et al. (2014) showed that the telomeres might even be longer in the mutants. This apparent discrepancy could be addressed using recent high-resolution methods for telomere length measurement (Lai et al., 2018; Kahl et al., 2020; Luo et al., 2020). More recently, RTEL1 was found to compensate telomere shortening in somatic cells in the absence of telomerase or replication protein A in *Arabidopsis* mutants (Olivier et al., 2018; Aklilu et al., 2020). Instead of knockout mutants and RNAi downregulation, RTEL1 domain specific mutants could be generated using CRISPR-based gene editing or TILLING populations (Schreiber et al., 2019) helping to fully characterise the functions of RTEL1 in both somatic and meiotic phases.

It is possible that there is some redundancy in recombination control as RTEL1 can rescue yeast *srs2* (suppressor of RAD Six-screen mutant 2) mutants in the presence of methyl methanesulfonate (Frizzell et al., 2014). SRS2 is another helicase that was shown recently to be involved in meiosis by preventing late prophase DNA damage in yeast (Sasanuma et al., 2019) and so it would be interesting to study its potential RTEL1 complementation in plants. *In vitro* biochemical characterisation of the *Arabidopsis SRS2* homolog demonstrated its 3′- to 5′-helicase activity and its involvement in processing different recombination intermediates (Blanck et al., 2009). Barley *SRS2* (BAnTr.GP.2HG007838, HORVU2Hr1G051300) is expressed in different tissues including anthers and meiocytes (Barakate et al., 2021; Milne et al., 2021) but its role in meiosis remains to be studied.

In conclusion, this study revealed the role of RTEL1 helicase in plant meiosis and control of recombination for the first time. The downregulation of *RTEL1* expression specifically in reproductive tissues impacted on recombination distribution but not overall rates. This effect is unlike the phenotype observed in *C. elegans* but may relate to the choice of knock-down mutants in this study as much as to the radically different genome architectures of the two species.

## Data Availability Statement

The SNPs data presented in the study are deposited in the European Variation Archive repisotory, accession number PRJEB47529 (Project) and ERZ3471485 (Analyses).

## Author Contributions

AB, CH, and RW contributed to experimental design. AB and SV contributed to RNAi construct preparation, screening and crosses of transgenic plants, and material collection. DD and JS produced the transgenic plants. MM, MA, and LR performed and analysed KASP and 50K genotyping data. MS performed expression data analysis. JO contributed to phylogenetic tree preparation. AB, MA, and RW contributed to manuscript preparation. All authors contributed to the article and approved the submitted version.

## Funding

This work was funded by the European Research Council (ERC Shuffle Project ID: 669182) and Biotechnology and Biological Science Research Council grant BB/F020872/1 and supported by the Scottish Government’s Rural and Environment Science and Analytical Services Division work programme Theme 2 WP2.1 RD1 and RD2.

## Conflict of Interest

The authors declare that the research was conducted in the absence of any commercial or financial relationships that could be construed as a potential conflict of interest.

## Publisher’s Note

All claims expressed in this article are solely those of the authors and do not necessarily represent those of their affiliated organizations, or those of the publisher, the editors and the reviewers. Any product that may be evaluated in this article, or claim that may be made by its manufacturer, is not guaranteed or endorsed by the publisher.

## Footnotes

^1^ http://www.bract.org/constructs.htm#barley
