# Supplementary material for: Downregulation of Barley Regulator of Telomere Elongation Helicase 1 Alters the Distribution of Meiotic Crossovers
Source: Front Plant Sci. 2021 Sep 30;12:745070. doi: 10.3389/fpls.2021.745070 (PMC8515186; doi:10.3389/fpls.2021.745070)
Supplement: Supplementary file 1 [file Data_Sheet_1.docx]

Supplementary Material

# Supplementary methods

Supplementary Method 1: Regeneration and characterization of *HvRTEL1*^RNAi^ transgenics.


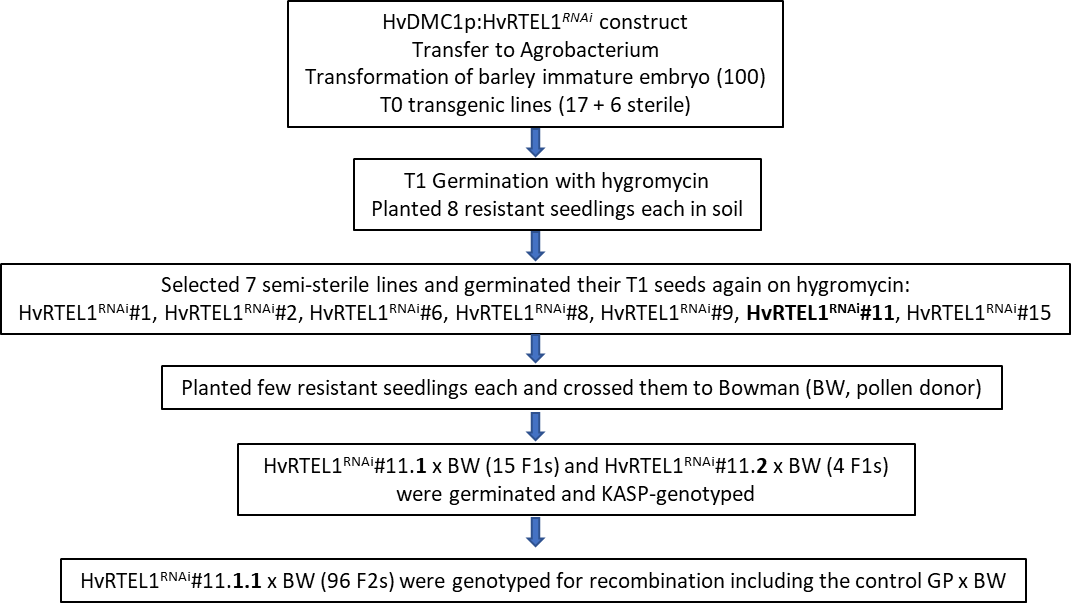


# Supplementary Data

## Supplementary Figures


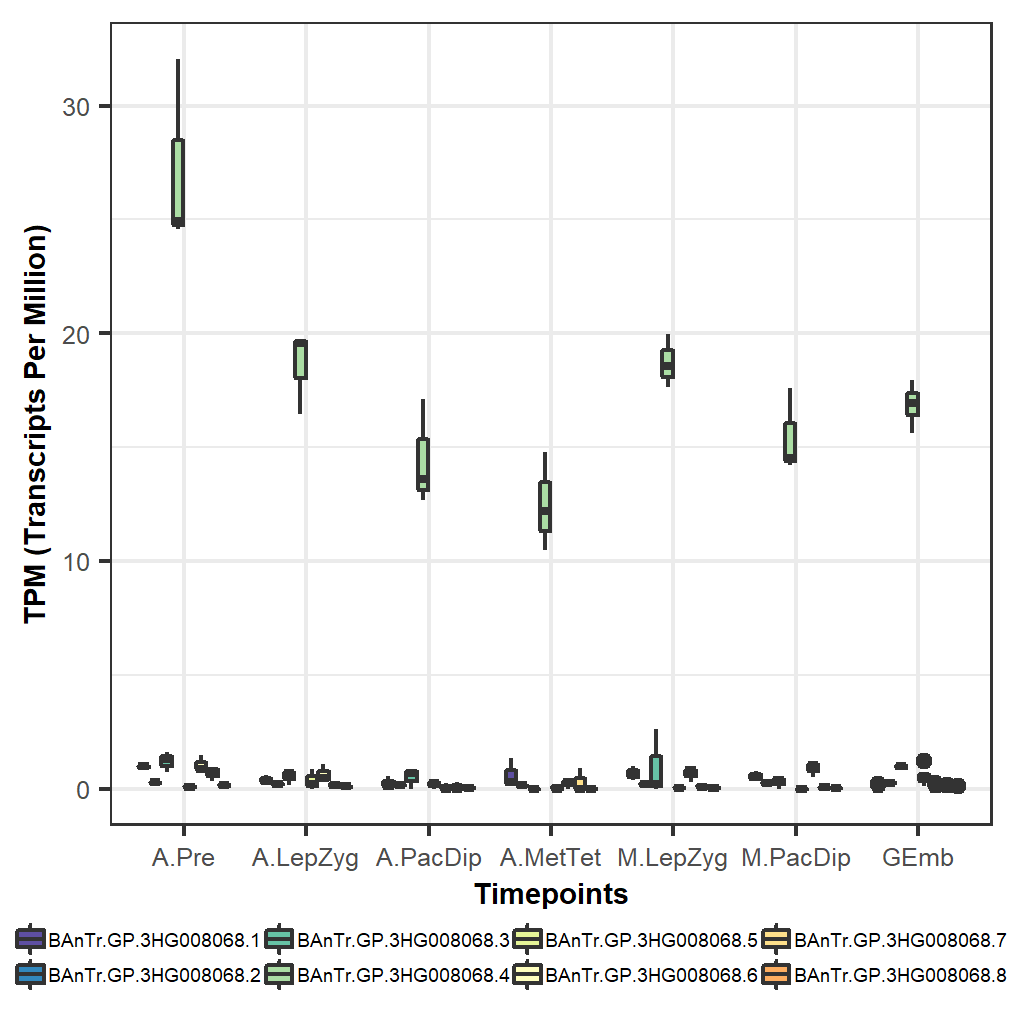


**Supplementary Figure 1:** Expression of different *RTEL1* isoforms.

Expression levels in barley anthers and meiocytes at different developmental stages. The samples (3 replicates each) are: A.Pre, anther pre-meiosis; A.LepZyg, anther leptotene–zygotene; A.PacDip, anther pachytene–diplotene; A.MetTet, anther metaphase I–tetrad; M.LepZyg, meiocyte leptotene–zygotene; M.PacDip, meiocyte pachytene–diplotene and Gemb, germinating embryos. The prefixes A. and M. in the sample names depict anther and meiocyte samples, respectively.

**Supplementary Figure 2**: RTEL1 phylogenetic tree.

The longest orthologous amino acid sequences from each species were used to build the maximum likelihood phylogenetic tree. The support values near the branches represent the percentages of replicate trees in which the associated taxa clustered together in the bootstrap test.


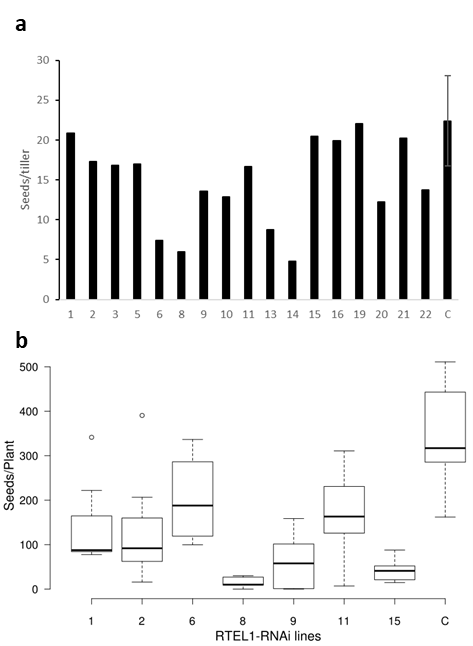


**Supplementary Figure 3:** Effect of RTEL1 down-regulation on fertility in barley.

**a)** Number of seeds per tiller of T0 *RTEL1*^RNAi^ lines. **b)** Average total number of seeds per plant of T1 *RTEL1*^RNAi^ lines. The control **(c)** is *GUS*^RNAi^ lines.


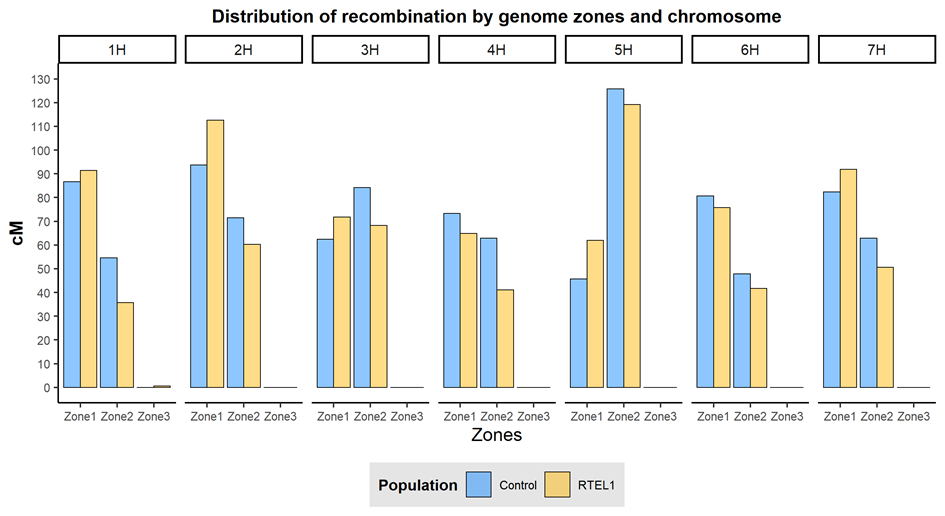


**Supplementary Figure 4:** Effect of *RTEL1*down-regulation on meiotic recombination at sub-chromosomal level.

Recombination distribution by genomic zone for the control GP x BW (blue) and *RTEL1*^RNAi^ x BW (yellow) populations per chromosome. The genomic physical limits between the genomic zones are described in Mascher et al., 2017. Significance refers to the Wilcoxon’s signed rank test being ns=not significant, * P<0.05, **P<0.01.

## Supplementary Tables

**Supplementary Table 1:** KASP markers used in this study.

| **JHI KASP** | **Chromosome** | **Location** | **Genotyping** |
| --- | --- | --- | --- |
| **11_10317** | 2H | 342925311 | F_1_ |
| **11_11243** | 7H | 601974427 | F_1_ |
| **SCRI_RS_214559** | 1H | 39417646 | F_2_ |
| **11_21357** | 1H | 334973336 | F_2_ |
| **11_20858** | 3H | 952287 | F_2_ |
| **12_31409** | 3H | 4853925 | F_2_ |

**Supplementary Table 2:** Recombination analysis using KASP markers.

|  | **1H pericentromeric** | **3H distal** |
| --- | --- | --- |
| SNPs | SCRI_RS_214559 (45 cM)  11_21357 (54 cM) | 11_20858 (1 cM)  12_31409 (8 cM) |
| GP x BW | 6.90 | 7.70 |
| *RTEL1*^RNAi^#11 x BW | 3.30 | 14.50 |

The recombination rate is in %. The SNP genetic location is indicated in cM.

**Supplementary Table 3:** Polymorphic marker summary.

The polymorphism start and end refer to where in the chromosome the first and last polymorphic marker is found, and the distal region not covered refers to the percentage of chromosome in both distal ends not covered by the polymorphism.

| **Chromosome** | **1H** | **2H** | **3H** | **4H** | **5H** | **6H** | **7H** |
| --- | --- | --- | --- | --- | --- | --- | --- |
| Chromosome size (Mbp) (Morex V2) | 522 | 675 | 628 | 624 | 599 | 573 | 634 |
| n. of Polymorphic markers | 772 | 1939 | 1834 | 1144 | 1877 | 1585 | 1974 |
| Zone 1: n. markers | 320 | 765 | 497 | 335 | 470 | 289 | 773 |
| Zone 2: n. markers | 448 | 1148 | 1336 | 800 | 1360 | 1176 | 990 |
| Zone 3: n. markers | 3 | 25 | 0 | 8 | 46 | 119 | 210 |
| Zone 1: SNP/Mbp | 5.9 | 10.2 | 7.9 | 8.6 | 12.0 | 6.9 | 11.6 |
| Zone 2: SNP/Mbp | 1.4 | 2.5 | 3.2 | 1.8 | 3.1 | 2.9 | 2.3 |
| Zone 3: SNP/Mbp | 0.0 | 0.2 | 0.0 | 0.1 | 0.4 | 1.0 | 1.6 |
| Chromosome start NOT covered | 0.03% | 0.02% | 0.03% | 0.03% | 0.33% | 0.26% | 0.24% |
| Chromosome end NOT covered | 0.10% | 0.00% | 1.12% | 0.35% | 0.00% | 0.02% | 0.00% |
| Total distal region NOT covered* | 0.13% | 0.02% | 1.15% | 0.38% | 0.33% | 0.28% | 0.24% |

**Supplementary Table 4:** Oligonucleotides used in this study.

| **Target gene** | **Oligonucleotide^a^** | **5’-Sequence-3’ ^b^** |
| --- | --- | --- |
| **RTEL1** | attB1-HvRTEL1-Ri | GGGGACAAGTTTGTACAAAAAAGCAGGCTACTCAAAGTATGGGGAAGTAGTTC |
| **RTEL1** | attB2-HvRTEL1-Ri | GGGGACCACTTTGTACAAGAAAGCTGGGTTGAACCCTTCAAGAAGTGTGAGTC |
| **Hygromycin** | HygF | TTGCATCGGCCGCGCTCCCGATTC |
| **Hygromycin** | HygR | TCGACCCTGCGCCCAAGCTGCATC |
| **β-glucuronidase** | attB1-GUS-Ri | GGGGACAAGTTTGTACAAAAAAGCAGGCTCTGTACAGCGAAGAGGCAGTCAAC |
| **β-glucuronidase** | attB2-GUS-Ri | GGGGACCACTTTGTACAAGAAAGCTGGGTCGTGGTGGTGGTGGTGGTGGCTAG |

^a^ Ri depicts oligos used for RNAi constructs. ^b^ Gateway attB1 and attB2 sequences are underlined.
